# Causal responsibility based explainable AI for vibrational spectroscopy applied to oral FTIR and oesophageal Raman diagnostics

**DOI:** 10.1038/s41598-026-62343-3

**Published:** 2026-07-18

**Authors:** Nathan Blake, David A. Kelly, Sarah Kapllani-Mucaj, Rong Wang, Geraint Thomas, Hana Chockler

**Affiliations:** 1 Department of Medical Physics and Biomedical Engineering, University College London, Gower Street, WC1E 6BT London, UK; 2 Department of Informatics, King’s College London, The Strand, WC2R 2LS London, UK; 3 Department of Cell and Developmental Biology, University College London, Gower Street, WC1E 6BT London, UK; 4 School of Dentistry, University of Missouri–Kansas City, 650 E 25th Street, MO 64108 Kansas City, Missouri USA

**Keywords:** Explainable AI, Vibrational spectroscopy, Raman spectroscopy, FTIR spectroscopy, Medical AI, Molecular diagnostics, Cancer, Computational biology and bioinformatics, Mathematics and computing

## Abstract

Biomedical applications of vibrational spectroscopy increasingly use deep learning, but these models often give classifications without revealing which spectral features drive their decisions. We introduce Spec-ReX, a causal responsibility-based explainable AI method for vibrational spectroscopy, and compare it with the widely used explainability methods SHAP and Grad-CAM across synthetic spectra, biochemical mixtures, oral FTIR tissue spectra and oesophageal Raman tissue spectra. In a definitive *in silico* experiment with known discriminative peaks, Spec-ReX achieved the highest ground truth localisation, with an Intersection over Union of 0.10 and attribution concentration of 0.56, compared with 0.09 and 0.26 for the next best method. In an ambiguous *in silico* experiment, SHAP achieved the highest positive hit rate, while Spec-ReX was most robust to removal of low-importance regions, consistent with a sensitivity–specificity trade-off. In the *in vitro* biochemical experiment, Spec-ReX was again the most robust to removal of low-importance regions, while SHAP performed best when removing high-importance regions. In the *ex vivo* datasets, where no definitive attribution ground truth was available, the methods returned different and architecture-dependent attribution patterns. Overall, Spec-ReX provides sparse and specific model-causal responsibility maps for spectral classifiers. These explanations pertain to trained model behaviour rather than biological causality, and their clinical utility remains to be validated through user-centred studies.

## Introduction

The use of AI in vibrational spectroscopy is rapidly increasing^1^, including biomedical applications such as cancer diagnostics^2^. This is driven by the successes of deep learning (DL) models across numerous biomedical domains. However, these models are often inscrutable: even when test performance is high, it can be difficult to determine why a classification was made. This is known as the ‘black-box’ problem.

There are both clinical and regulatory pressures for making black-box AI more transparent. Across multiple clinical areas, clinicians generally deem it essential for establishing trust in AI^3–7^. Regulation, such as the EU AI Act, similarly requires high-risk AI systems to be sufficiently transparent for deployers to interpret their outputs^8^. For biomedical spectroscopy, this creates a practical requirement: it is not enough to report a high classification accuracy, it is also necessary to understand the features driving model performance.

We use the term *interpretability* for models whose behaviour is intrinsically understandable, such as linear models in which coefficients have a direct meaning. We use *explainability* for methods that attempt to explain otherwise inscrutable models after training. Post-hoc explainable AI (XAI) methods may be local or global, model-agnostic or model-specific, and may operate through feature attribution, counterfactual reasoning or example-based comparison^9,10^. This taxonomy is useful here because vibrational spectroscopy requires local, feature-level explanations: the relevant question is often which wavenumber regions caused a model to assign a spectrum to a particular class.

There is therefore a need for domain-specific XAI methods and for the systematic evaluation of their behaviour on spectral data. Common XAI methods such as Shapley Additive Explanations (SHAP)^11^ and Gradient-weighted Class Activation Mapping (Grad-CAM)^12^ are designed for generic use, but some spectral adaptations have been proposed. Quotient Game was developed to make SHAP more computationally efficient for Raman spectroscopy^13^, while zones-based SHAP splits spectra into more interpretable regions before applying SHAP^14^. Spec-ReX extends ReX (causal Responsibility eXplanations) to vibrational spectra^15^. It uses the formal theory of actual causality^16^ to identify spectral intervals responsible for a model’s output.

It is important to note that XAI methods explain the trained model and the input spectra. They do not establish biological causality, nor do they prove that a highlighted band is a causal disease mechanism. This distinction is important. A poor or shortcut-driven model can still be faithfully explained; indeed, explanations may be valuable precisely because they reveal brittle or biochemically implausible model behaviour that conventional performance metrics obscure.

In this paper we compare Spec-ReX with gradient SHAP and Grad-CAM. There are numerous SHAP variants; we use gradient SHAP (hereafter SHAP) because it is common in the Raman literature and has shown strong performance on one-dimensional datatypes^17^. The evaluation proceeds in three stages. First, we use realistic *in silico* datasets; one with a definitive and one with an ambiguous ground truth signal. Second, we use *in vitro* Raman spectra from biochemical mixtures, retaining partial control over the data-generating process while introducing real-world spectral complexity. Third, we assess two *ex vivo* datasets: FTIR spectra from oral tissue and Raman spectra from oesophageal tissue. This structure explicitly separates controlled methodological validation from exploratory biomedical applicability. The *in silico* experiments test whether the explainers can recover known model-relevant features; the *ex vivo* experiments explore how the methods behave when no definitive ground truth is available.

A critical difficulty in assessing post-hoc XAI tools is that it is difficult to disentangle the AI model and the XAI tool. A poorly trained model may result in unrealistic explanations, but this is a feature of the model, not the XAI tool. A poor XAI tool applied to a ‘good’ model may also result in unrealistic explanations, but that then is a feature of the XAI tool.

## Causal responsibility explanations

Spec-ReX is an adaptation of an XAI tool called ReX^18^. It is based on the mathematical framework of actual causality^16^ and quantifies the degree of *causal responsibility*^19^ each feature has towards a model’s classification. Throughout this paper, causal responsibility is defined with respect to the trained model under the masking intervention.

***Responsibility***

Causal responsibility measures the importance of a cause towards an effect^19^. We use the definition provided by Chockler et al.^18^. Let $x \in \mathbb {R}^{T}$ be a spectrum sampled on *T* wavenumbers, and let $\mathcal {N}$ be a trained classifier with prediction $o = \mathcal {N}(x)$. Let $\Pi _i = \{I_{1},\dots ,I_{s}\}$ be a partition of the index set $\{1,\dots ,T\}$ into *s* disjoint contiguous intervals. For a set of intervals $S \subseteq \Pi _i$, let $\textrm{mask}(x,S)$ denote the spectrum obtained by replacing the intensities on $\bigcup _{I\in S} I$ by a ‘neutral’ value (discussed later).

Then the *degree of responsibility* $r(I_i,x,o)$ of $I_i$ for *x* being classified as *o* is defined as *1/(k+1)*, where *k* is the size of the smallest *witness set* $I_j$ for $I_i$. If keeping $I_{i}$ at the original value and setting all other parts to the neutral value maintains the prediction, then *k=0* and $r(I_{i},x,o)=1$. If all segments are required, then it suffices to change one segment to flip the classification. If there are 4 segments, for example, then the cardinality of the witness set is *k = 3* and the responsibility is then *1/(3+1)* or 0.25 for each segment.

For each segment or combination of segments with non-0 responsibility, the process of partitioning and responsibility calculated is repeated. The process of iterative refinement ends when a pre-defined search budget is exhausted or no segment in the current partition has the required classification. Pseudocode for the algorithm is provided in supplemental information A.

**Fig. 1:**
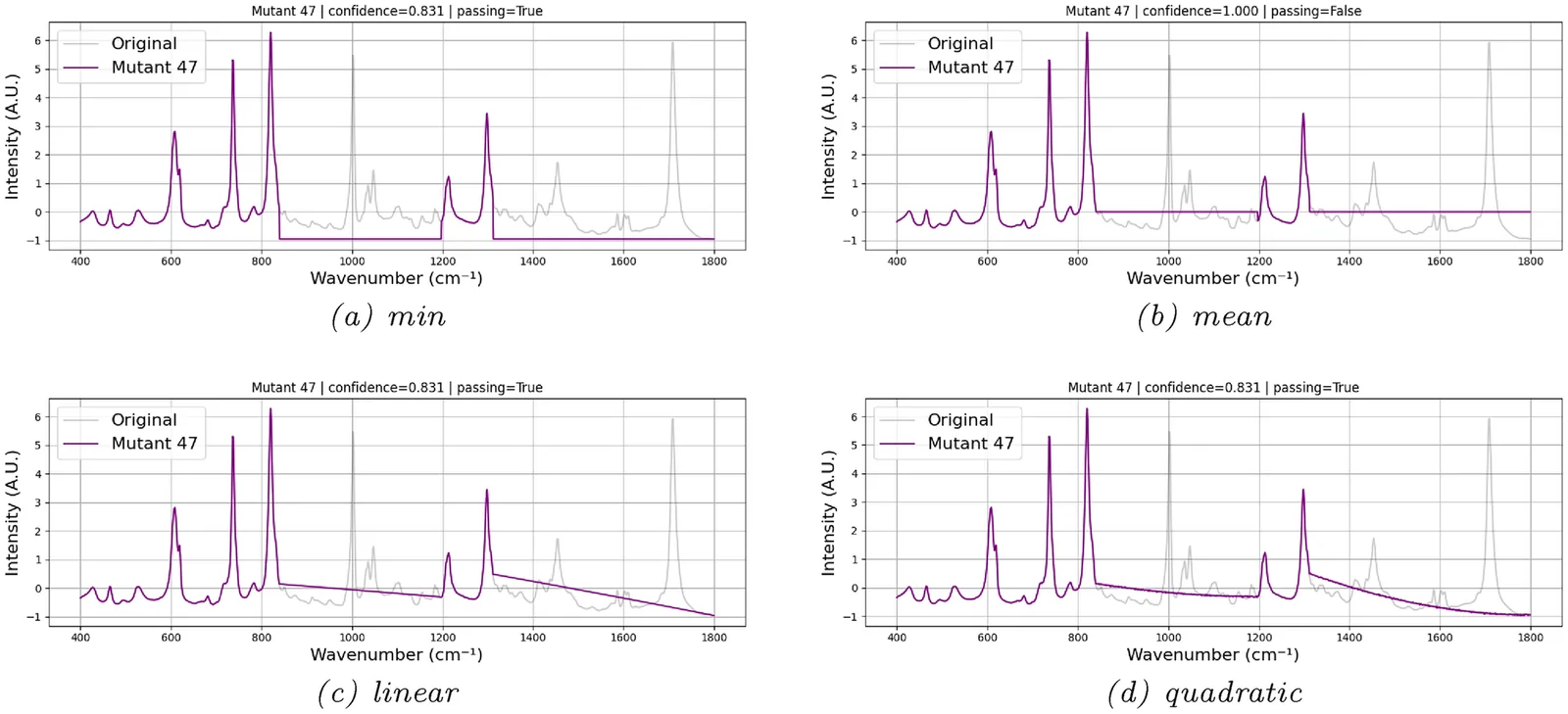
Spec-ReX mutants produced by four occlusion methods: min, mean, linear and quadratic. Purple spectrum is the mutant spectrum, grey spectrum the original. Zero is another common default perturbation, but for this dataset coincides with the mean as the spectra have been normalised to have zero mean.

Neutral masking values are a critical aspect of perturbation based XAI methods: they should remove spectral features while minimising out-of-distribution (OOD) artifacts. In Spec-ReX, the ‘neutral’ replacement values, $\textrm{mask}(x,S)$, are given by joining the ends of each masked segment with a quadratic, with an adjustable degree of Gaussian noise added. This makes for more realistic perturbations (called mutants in ReX) compared to common default values used in perturbation XAI methods such as minimum, mean or linear interpolation, which are optionally available in Spec-ReX (Fig. 1). ReX explanations are causal with respect to the model and input features - it asks which features cause a model to give a particular output.

Like SHAP and Grad-CAM, Spec-ReX is a post-hoc XAI tool. These highlight which features the model used to reach a given classification. In this sense, they provide local explanations (i.e. highlighting features of a single input or spectrum), as opposed to global explanations (i.e. highlighting features generally used by a model). SHAP and Spec-ReX are model agnostic in that they can be applied to any model. Gradient SHAP, however, requires access to the model gradients. Grad-CAM is limited to convolutional neural networks (CNNs), which dictates the granularity of its output. The outputs of SHAP and Grad-CAM are often referred to as saliency maps. Spec-ReX outputs responsibility maps. These are very similar, and for most purposes can be regarded as attributing importance to inputs. We therefore refer to the collective outputs of these XAI tools as *attribution maps*.

## Methods

Assessing the correctness of an XAI attribution map is referred to as the fidelity of the explainer^10^; also called its faithfulness^20^. We assess fidelity using complementary strategies rather than a single score. When ground truth is known, attribution maps can be compared directly with known discriminative spectral regions. When ground truth is ambiguous or absent, fidelity must be inferred indirectly by measuring how model predictions change when features ranked as important (or unimportant) are inserted or removed.

Inspecting local explanations on every single spectrum in an entire dataset is prohibitive. Thus, to provide class-wise information, from each XAI tool we take the mean attribution map per class to find those features which the model used on average.

For all experiments, standard-normal variate normalisation was performed. No further pre-processing (e.g. baseline correction or smoothing) was undertaken. As the models themselves are not the object of study, no hyper-parameter optimisation was undertaken. All model performance metrics (i.e. accuracy) pertain to the test set, as are all XAI performance measures.

**Fig. 2:**
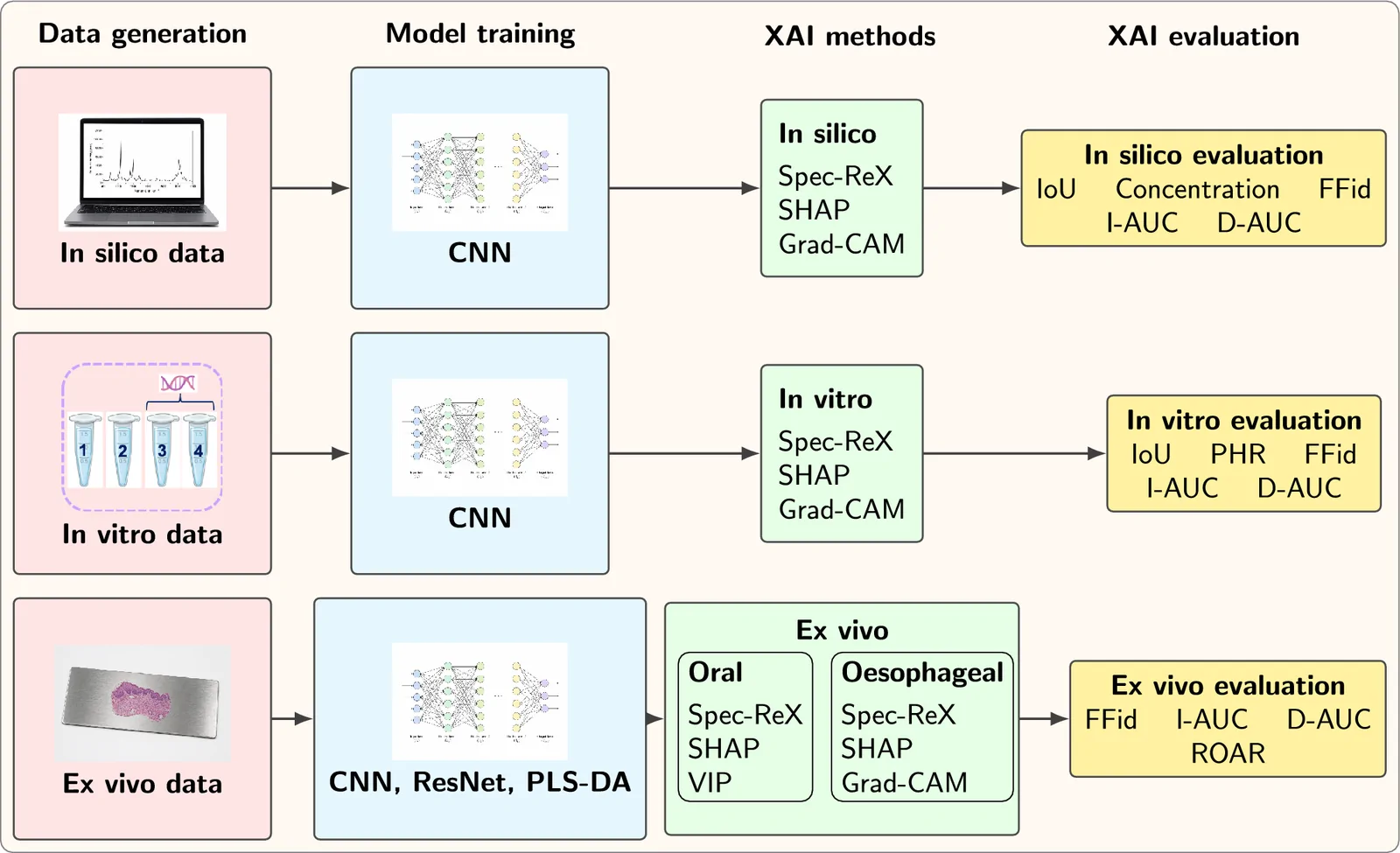
Schematic overview of the experimental design. In silico, in vitro, and ex vivo datasets are independently generated, used for machine learning model training, XAI applied to each date, and evaluated using quantitative XAI performance metrics.

A schematic overview of the experiments is shown in Fig. 2. The study deliberately progresses from controlled methodological validation to biomedical application. The *in silico* experiments provide the strongest test of XAI fidelity because the discriminative signal is known by construction. The *in vitro* experiment retains partial biochemical control but introduces real spectral variation. The *ex vivo* datasets are the most clinically relevant, but they do not provide any ground truth for attribution fidelity. Results from these datasets are therefore interpreted as exploratory evidence of model behaviour rather than as validation of disease biology.

### In silico data

Spec-ReX has been assessed under simulated conditions, demonstrating that it faithfully locates to only those features which the model was forced to learn^15^. However, this *in silico* simulation was too simplistic to represent biomedical spectroscopy under realistic conditions. Therefore, here we create two additional *in silico* datasets, one with a definitive and one with an ambiguous ground truth.

In both cases a spectral library of pure biochemical samples was utilised (details in supplementary information B). Each spectrum in the spectral library is called an endmember.

For these datasets we consider only the positive classes. Explaining the absence of features (i.e. the null class) is a far less developed field of research within the XAI literature^21^.

***In silico: definitive ground truth***

From the spectral library we took the endmembers: di–C$_8$ phosphatidylcholine, Phenylalanine, Lactate and Collagen, with Mucin in half the samples. These were linearly combined with equal weighting. Gaussian noise was added. Random wavenumber shifting of up to 5 wavenumbers was induced. 4000 spectra were created, which constitutes class 0, the null class. Class 1 was similarly created, except an artificial peak was added at 783 $cm^{-1}$. Class 2 had two peaks added at 497 and 1485 $cm^{-1}$ and class 3 peaks at 1099, 1337 and 1375 $cm^{-1}$.

With this data we trained a small CNN, previously used in cancer diagnostics^22^, to 99.9% accuracy. SHAP, Grad-CAM and Spec-ReX were then applied to every spectrum in the test set. Spec-ReX was run with four masking methods and SHAP with three. With a definitive ground truth, we explore the role of masking values in both Spec-ReX and SHAP. Spec-ReX utilises minimum, mean, linear and quadratic. We use the standard SHAP variants of the spectrum’s mean, a random contra-labeled spectrum and the dataset mean.

***In silico: ambiguous ground truth***

From the spectral library we created four ’conditions’ as given in Table 1. The latter two conditions contain DNA in addition to all those contained in the former two. Each condition had 4000 spectra which were evenly-weighted linear combinations of the endmembers with Guassian noise, spectral shifting and random peak injection. We then built a binary classifier for ’no DNA’ or ’with DNA’.

This creates an ’ambiguous’ ground truth in the sense that, although we know that the only difference between the conditions is DNA, we cannot control precisely which of the multiple DNA associated peaks the model will learn. This is far more indicative of real world biomedical spectra.

### In vitro data

Using the same setup as the *in silico* ambiguous ground truth experiment, we created an *in vitro* dataset. This involved the deposition of laboratory standard biochemicals which were then interrogated by Raman spectroscopy. We chose DNA as it is sufficiently complex to represent a challenge for DL as well as being implicated in various cancer pathogenic pathways (e.g. polyploidy), making it a relevant target. While this does provide a ground truth signal (i.e. DNA associated peaks), it is even more ambiguous than the corresponding *in silico* datasets as conformational changes can induce peak shifting.

21mg/ml stock solutions of each biochemical were prepared in nuclease-free H_2_O. Four mixtures were prepared as depicted in Table 1, with the proportion of DNA kept constant in each sample. Four technical replicates of each sample were deposited onto a steel slide at 1 µl per spot and left to dry under a fume hood for 24 hours.

Raman spectra of the biochemical samples were acquired on a Renishaw Raman Invia microscope using step scans and extended scan mode with 785 nm laser excitation at 50% laser power, with one accumulation and five seconds exposure per point. Spectra were collected in 350–1850 $cm^{-1}$. With this setup, the laser beam was focused onto the sample and scattered light was collected with a 20x objective lens. Grid maps of approximately 1000 spectra per sample were acquired. Renishaw’s LiveTrack feature was used to compensate for variations in sample thickness. Inelastically scattered light was collected and diffracted by a 1800 grooves/mm diffraction grating. Cosmic rays were removed automatically by median filtering using Renishaw’s WIRE software. A total of 16107 spectra were collected.

From this we created two classes: ’no DNA’ consisting of condition 1 and condition 2, and “with DNA” consisting of condition 3 and condition 4. We supplemented this with a ’dust-bin’ class, which allows models to assign ’junk’ inputs. This has been shown to improve performance^23^. This involved taking the steel-slide endmember from the spectral library and perturbing the spectrum with Gaussian noise, wavenumber shifting and random peak insertion. 4000 spectra were thus created. A 3 class-CNN was then trained using leave-one-replicate-out cross-validation (CV), achieving 93.3% accuracy.

**Table 1:** Biochemical composition of Conditions 1–4.

Condition	Biochemical Spectra
Condition 1	di–C$_8$ phosphatidylcholine, Phenylalanine, Lactate
Condition 2	di–C$_8$ phosphatidylcholine, Phenylalanine, Lactate, Collagen, Mucin
Condition 3	di–C$_8$ phosphatidylcholine, Phenylalanine, Lactate, DNA
Condition 4	di–C$_8$ phosphatidylcholine, Phenylalanine, Lactate, Collagen, Mucin, DNA

### Ex vivo data

We further assess Spec-ReX on two previously published vibrational datasets for cancer diagnostics. These represent the most clinically relevant datasets, but lack any sense of a ground truth beyond biochemical plausibility. Both datasets followed the Ethical Principles and Guidelines for the Protection of Human Subjects of Research in accordance with an approved ethical proposal [Gloucestershire Local Research Ethics for the Oesophageal dataset and the Committee and Institutional Review Board of the University of Missouri–Kansas City for the Oral dataset], in which patients gave informed consent for their tissues being used for future research. Unlike the previous experiments, these datasets are not used to prove that an explainer has recovered a known signal. They are used to examine how the methods behave when applied to clinically motivated spectral classifiers and to determine whether the returned attributions are sparse, stable across architectures, and biochemically plausible.

***Oesophageal cancer dataset***

This data was originally published in a study investigating cross instrument/institution transferability with a clinically motivated 5-class classifier^22^. Briefly, this involved obtaining Raman spectra from matched samples on three distinct spectrometers. To facilitate the interpretation of the attribution maps, for this paper we trained two new binary models. The first of these models has the same architecture as the original paper, but with just two classes (normal squamous vs high grade dysplasia and adenocarcinoma). The model was trained on data from two instruments and tested against the held-out instrument data. This achieved 89.7% accuracy. A larger ResNet model, pre-trained on a bacterial Raman dataset^24^, was similarly trained on the same data. This achieved 81.5% accuracy. This allows us to assess XAI consistency of across model architectures.

***FTIR oral cancer dataset***

This data was originally published in a study to assess the suitability of FTIR spectroscopy to distinguish malignant from benign oral tissue^25^. Two representative epithelial spectra were obtained per sample. As this results in relatively few spectra, a more traditional machine learning (ML) technique was used. Partial-least squares - discriminant analysis (PLS-DA) with 3 components achieved an accuracy of 93.48%.

As Grad-CAM is a CNN specific XAI method, it cannot extract attribution maps from a PLS-DA model. Variable Influence on Projection (VIP) is an interpretability layer used to assign importance to features used in least squares frameworks^26^. It produces global importance estimates. This gives a natural comparison to the mean attribution maps calculated using Spec-ReX and SHAP, in place of Grad-CAM.

### Quantifying performance

Here we describe the XAI performance metrics used in each section. With the definitive ground truth, *G*, of section 3.1, we can quantitatively assess XAI attribution maps. We use the intersection over union (IoU) and concentration. The former requires a boolean attribution mask, $A_b$, which we create by assigning true when the attribution map was above 0.5.

$$\textrm{IoU}(A_b,G) \;=\; \frac{|A_b \cap G|}{|A_b \cup G|}.$$

The concentration quantifies the fraction of attribution mass, *A*, inside the ground truth regions.

$$\textrm{Concentration}(A,G) \;=\; \frac{\sum _{j \in G} A_j}{\sum _{j=1}^{L} A_j}.$$

For the ambiguous ground truth of sections "In Silico Data" and "In Vitro Data" we make the assumption that the DNA associated peaks are the ground truth (though the model may not have learned to identify all these peaks). We take these peaks as point estimates (i.e. a single wavenumber) of the ground truth. This means the concentration is no longer defined. We thus include the positive hit rate (PHR), which measures how many known reference peaks are “hit” by an XAI attribution region. Additionally, for the IoU, we assume a delta of one wavenumber around the peak to calculate a score.

For the *ex vivo* data there is no known ground truth signal, hence there is no unambiguous method for quantitatively assessing XAI methods^10^. We instead utilise methods which assess model performance when important or unimportant attributions are inserted or deleted. Insertion and deletion area under characteristic curves^27^ (iAUC and dAUC respectively) are the most commonly used method^28^, and have been noted to translate well to 1D datatypes^17,29,30^. Insertion curves are based on the premise that if features are important, then (starting from some baseline) incrementally adding them should rapidly converge on the required classification. Similarly, deletion curve performance should yield drops in performance as important features are deleted. These features are inserted or deleted based on the attribution map.

This necessitates a choice as each feature needs to be inserted or deleted according to some baseline or default value. The default is a zero baseline, but to ensure that results are not overly sensitive to the baseline choice we additionally include a baseline from the dataset mean, a random spectrum from the other class and the other class average. For brevity we report the mean of all baselines, though full results from some experiments are available in the supplementary information.

Insertion and deletion curves have been criticised, however, for inducing OOD perturbations. Remove and retrain (ROAR) overcomes this by retraining the entire model, having removed the top K% of attributions^31^. However, not only is this computationally expensive, but also induces data leakage (via the XAI method directing attention) and explains the retrained model rather than the original model. Fine-tuned Fidelity (F-Fidelity) attempts to overcome these drawbacks by fine-tuning the model rather than retraining it^32^. Although this will still be a different model, it will generally be closer to the original model than one fully retrained. Additionally it avoids data leakage by inducing XAI agnostic perturbations, which are then in-distribution for the fine-tuned model. F-Fidelity assesses feature importance by quantifying the drop in model performance when the most important features are removed (FFid$^{+}$) and the least important (FFid$^{-}$).

The exception to this is section 4.3 in which PLS-DA is applied. ’Fine-tuning’ is ill-defined for traditional models and so we revert to using ROAR. This includes using several baseline values (overall mean, opposite class mean and the default of zero). We take the mean of these.

The iAUC/dAUC and F-Fidelity are also applied to the earlier datasets, building an intuition of how these measures relate to a known ground truth. This will be useful later when the ground truth is more ambiguous, or not known at all.

## Results

Each XAI method varies in how it presents its outputs. Although arbitrary, we have kept these outputs as closely as possible to maintain qualitative impressions. However, we min-max normalised all attribution maps to the interval [0, 1] to facilitate downstream quantitative comparisons. The results are presented in order of decreasing experimental control: first *in silico* datasets with known or partially known discriminative structure, then *in vitro* biochemical spectra, and finally *ex vivo* tissue datasets where no known ground truth is available.

### In silico results

***In silico: definitive ground truth***

**Fig. 3:**
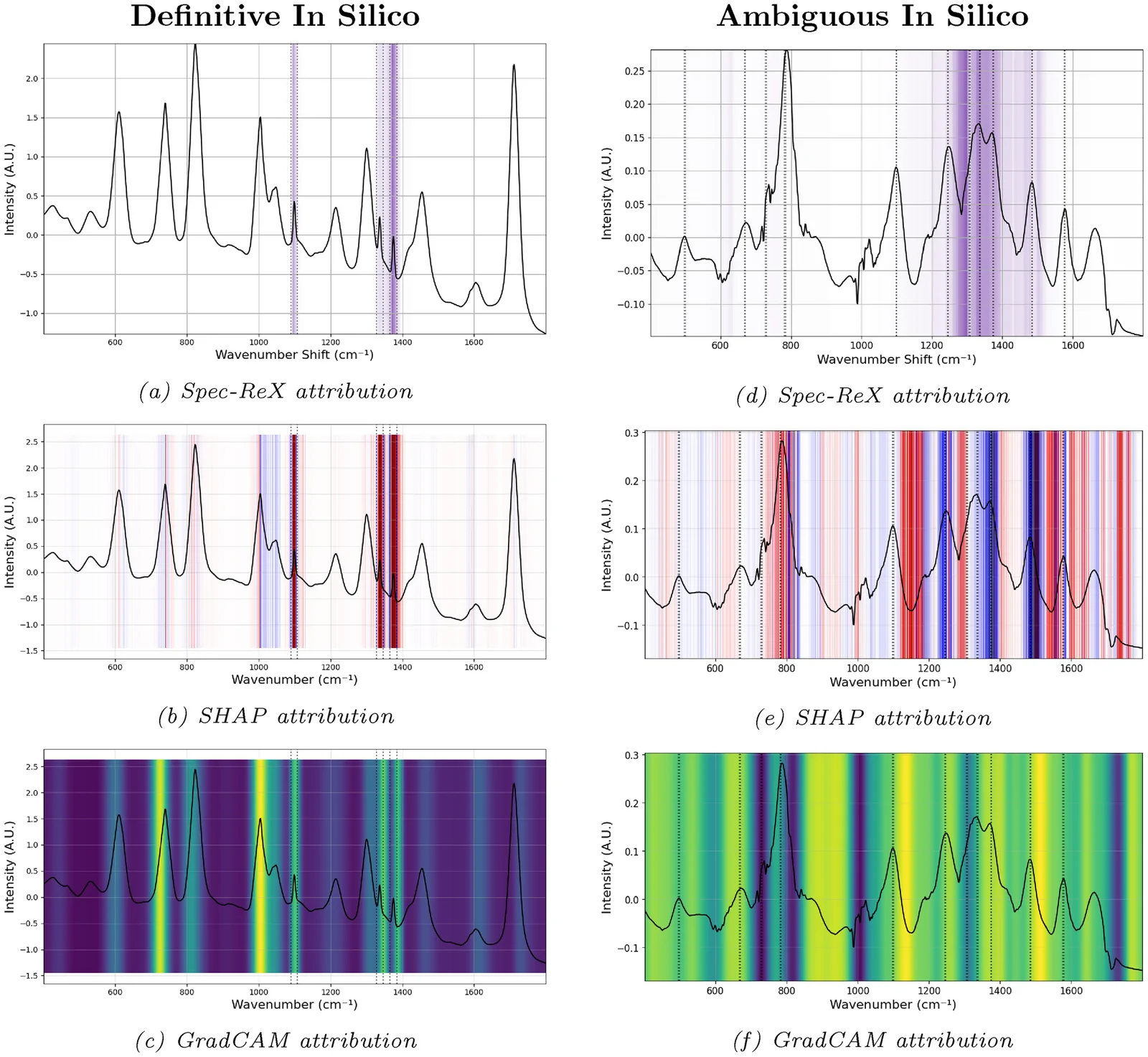
Left column (**a-c**): In Silico definitive data, mean spectra of class three (dotted vertical lines indicate defining peaks) with mean attribution maps. Right column (**d-f**): In Silico ambiguous data, difference spectrum.

Figure 3 (left column) shows the mean attribution maps for class 3, which is defined by three artificial peaks, plotted with the class mean spectrum. All three methods localise to the discriminating peaks. However, the attribution patterns differ substantially. Grad-CAM broadly identifies the relevant regions, but its attribution is slightly off-centre, consistent with the coarse spatial resolution imposed by the final convolutional layer. SHAP identifies the true peaks but also assigns attribution to many additional regions. Some of this signal reflects SHAP’s ability to indicate regions which would make the class less likely if present, but in this controlled experiment there are no such true negative-defining regions. These additional attributions therefore represent noise. Spec-ReX gives the sparsest attribution map, with most attribution mass concentrated around the class-defining peaks. Similar qualitative behaviour is seen across the other positive classes (Supplementary Fig. C.5).

The quantitative results support this visual interpretation (Table 2). The quadratic Spec-ReX variant achieves the highest IoU (0.10) and the highest concentration (0.56). The IoU improvement over the best SHAP variants is modest, but the concentration improvement is substantial: the next best concentration is 0.26 for GradSHAP-datasetAvg. This indicates that the primary advantage of Spec-ReX in this controlled setting is not only that it reaches the correct peaks, but that it places much less attribution mass outside them.

The metrics that do not use ground truth give a more mixed picture. ReX-min achieves the highest iAUC (0.76), while GradSHAP-randcontraclass and GradSHAP-datasetAvg achieve the highest FFid$^{+}$ (0.17). Quadratic Spec-ReX nevertheless remains competitive on dAUC (0.16) and achieves the best FFid$^{-}$ (0.00), indicating robustness to removal of regions ranked as low importance. The discrepancy between ground truth metrics and perturbation-based metrics is informative: i/dAUC and FFid measure how the trained model responds to feature insertion or deletion, whereas IoU and concentration measure localisation against known discriminative peaks. These quantities need not agree, even in a controlled setting.

The iAUC and dAUC values in Table 2 are averaged across four baseline strategies. The full baseline-specific results are shown in Supplementary Table 8. The ranking of methods is broadly preserved across baselines, indicating that the insertion/deletion conclusions are not driven by a single baseline choice.

Overall, the definitive *in silico* experiment shows that all three XAI methods can identify learned discriminative peaks when the ground truth is simple and known. Spec-ReX is the most specific method by ground truth concentration and FFid$^{-}$, while SHAP variants perform well on metrics that reward sensitivity to sufficient features. A full results table is available in supplementary information, table 7, which breaks down the results by class. For all subsequent analyses, we only include results from the ‘quadratic’ Spec-ReX variant and the ‘dataset average’ variant of SHAP.

**Table 2:** In Silico definitive results: iAUC (*↑*), dAUC (*↓*), FFid$^{+}$ (*↑*), FFid$^{-}$ (*↓*). Arrows indicate the direction of better performance. Best performing method per measure shown in bold.

Method	IoU	Concentration	iAUC	dAUC	FFid$^{+}$	FFid$^{-}$
GradSHAP-mean	0.09	0.18	0.25	0.19	0.06	0.07
GradSHAP-randcontraclass	0.09	0.24	0.22	**0.16**	**0.17**	0.15
GradSHAP-datasetAvg	0.09	0.26	0.21	**0.16**	**0.17**	0.16
Grad-CAM	0.01	0.01	0.43	0.47	0.05	0.02
ReX-quadratic	**0.10**	**0.56**	0.70	**0.16**	0.02	**0.00**
ReX-linear	0.07	0.44	0.67	**0.16**	0.02	0.01
ReX-min	0.07	0.09	**0.76**	0.26	−0.01	0.02
ReX-mean	0.07	0.22	0.72	0.20	0.02	0.04

***In silico: ambiguous ground truth***

The ambiguous in silico experiment moves from artificial class-defining peaks to a more realistic biochemical distinction. The classes differ by the presence of DNA, but the model is free to learn any subset of DNA-associated spectral features.

Figure 3 (right column) shows the class difference spectrum and the mean attribution maps for the positive DNA class. The difference spectrum highlights several DNA-associated peaks. Spec-ReX assigns strongest responsibility to regions around 1336, 1374 and 1419 $cm^{-1}$, corresponding to prominent features in the DNA endmember. The first two are associated with purine bases and ring breathing modes of DNA^33^. Spec-ReX also assigns responsibility around 1286 $cm^{-1}$, close to a cytosine-associated peak at 1287 $cm^{-1}$, and to lower-responsibility regions at 602–620 $cm^{-1}$ and 1158–1196 $cm^{-1}$, which are associated with nucleotide conformation and backbone or phosphate interactions^33^.

SHAP is most prominent at 1153 and 816 $cm^{-1}$, although these features are difficult to distinguish visually because of the dispersed attribution pattern. Grad-CAM is most prominent at 489, 558, 695 and 769 $cm^{-1}$, with the latter two regions associated with nucleotide conformation. Both SHAP and Grad-CAM also highlight regions around 783 $cm^{-1}$, which is the most prominent feature in the difference spectrum. The three methods therefore agree that DNA-associated regions are relevant, but they do not agree on which regions are most responsible for the model’s classification.

Table 3 quantifies this difference. SHAP achieves the highest PHR (0.31), iAUC (0.84), dAUC (0.52) and FFid$^{+}$ (0.0714), indicating that it is more sensitive to DNA-associated features. Spec-ReX achieves the highest IoU (0.05) and the lowest FFid$^{-}$ (0.0006), consistent with a more specific attribution pattern. Grad-CAM performs worst on IoU and PHR and is also weaker on the perturbation-based metrics.

**Table 3:** Ambiguous *In Silico* performance. Positive Class (Class 1) vs DNA peaks: IoU (*↑*), PHR (*↑*); iAUC (*↑*), dAUC (*↓*), FFid$^{+}$ (*↑*), FFid$^{-}$ (*↓*). Arrows indicate the direction of better performance.

Method	IoU	PHR	iAUC	dAUC	FFid$^{+}$	FFid$^{-}$
GradientSHAP	0.04	**0.31**	**0.84**	**0.52**	**0.0714**	0.0338
Grad-CAM	0.02	0.11	0.66	0.69	0.0002	0.0028
Spec-ReX	**0.05**	0.24	0.74	0.58	0.0012	**0.0006**

These results illustrate an important distinction between data explainability and post-hoc explainability. The difference spectrum identifies the spectral features that distinguish the two data distributions. The attribution maps identify the features used by the trained model. These need not be the same. In this experiment, the model appears to rely on only a subset of the available DNA-associated features. This is consistent with shortcut learning, in which a model learns a sufficient subset of class-discriminative features rather than all biologically relevant features^34^. However, biochemical plausibility alone is not proof of XAI fidelity, and plausible attribution maps can still be misleading if they do not reflect the model’s true reasoning^35^.

Overall, the ambiguous *in silico* experiment indicates a sensitivity–specificity trade-off. SHAP identifies more DNA-associated regions, whereas Spec-ReX is more localised. The disagreement between PHR, IoU, iAUC, dAUC and FFid also demonstrates why no single metric should be treated as definitive.

### In vitro results

**Fig. 4:**
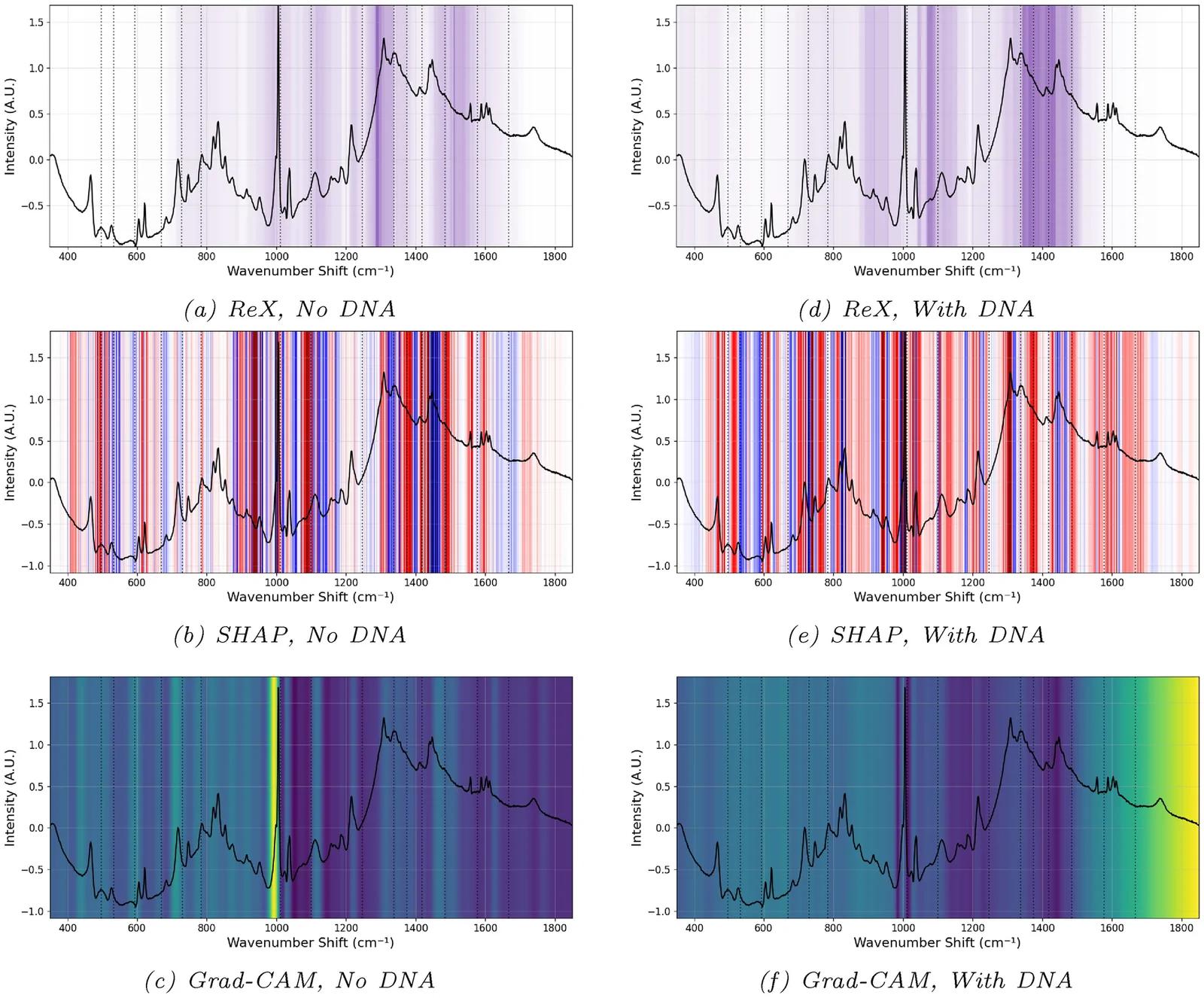
Difference spectra and mean attribution maps arranged by method (rows) and class (columns). Dotted vertical lines denote known DNA endmember associated peaks.

The *in vitro* experiment provides an intermediate level of realism; class distinction is still experimentally controlled, but the spectra are acquired from real biochemical deposits. Consequently, conformational changes, drying effects and peak shifts make the ground truth less precise than in the *in silico* experiments.

Figure 4 shows the difference spectrum and the mean attribution maps for the no-DNA and with-DNA classes. Unlike the definitive *in silico* experiment, the no-DNA class cannot be treated simply as a class defined by the absence of known peaks. Real spectra may contain shifted, overlapping or conformation-dependent features. This is reflected in the difference spectrum: the largest differences occur at 1006 and 1309 $cm^{-1}$, only the former of which is close to a DNA peak in the reference library.

For the with-DNA class, SHAP assigns its strongest importance to 1002 $cm^{-1}$, although the barcode-like attribution pattern makes this difficult to see visually. Grad-CAM assigns greatest importance to the high-wavenumber end of the spectrum, particularly above 1650 $cm^{-1}$, a region that includes the DNA-associated peak at 1667 $cm^{-1}$. Spec-ReX identifies two main regions, 1342–1437 $cm^{-1}$ and 1070–1084 $cm^{-1}$; the former includes DNA-associated peaks at 1375 and 1418 $cm^{-1}$. Thus, all three methods return biochemically plausible regions, but they emphasise different parts of the spectrum.

The quantitative results are shown in Table 4. For the with-DNA class, Grad-CAM achieves the highest IoU (0.0094) and PHR (0.3371) against known DNA-associated peaks. However, these ground truth comparisons assume that DNA peaks are neither shifted nor altered by the experimental context, and that the trained model uses the same reference peaks. The perturbation-based metrics do not support a simple interpretation in which Grad-CAM is the most faithful method: for class 1, Grad-CAM performs worse than Spec-ReX on iAUC and FFid$^{-}$, and worse than SHAP on dAUC. A full results table, is shown in supplementary Table 10.

Spec-ReX achieves the highest iAUC (0.63) and the lowest FFid$^{-}$ (0.0007), while SHAP achieves the lowest dAUC (0.38) and highest FFid$^{+}$ (0.0470). This again suggests a division between metrics that reward broad sensitivity to sufficient features and metrics that reward robustness to removal of low-importance regions. Grad-CAM performs poorly on the overall dAUC and FFid measures relative to SHAP and Spec-ReX.

The no-DNA class further illustrates the difficulty of interpreting attribution maps in realistic spectra. SHAP identifies 1001 $cm^{-1}$ as most important, which is close to the 1002 $cm^{-1}$ feature it identifies for the with-DNA class. This suggests that the model may be using a small peak shift around 1001 $cm^{-1}$ as a discriminating feature. Grad-CAM broadly agrees, highlighting 992 $cm^{-1}$. Spec-ReX assigns some responsibility to this region, but more strongly highlights 1287–1293 $cm^{-1}$ and 1509–1512 $cm^{-1}$, both associated with cytosine^33^. These patterns are biochemically plausible, but they should be interpreted as explanations of the trained classifier rather than as direct biochemical conclusions.

Overall, the *in vitro* results broadly reproduce the *in silico* sensitivity–specificity pattern, but with greater ambiguity. The ground truth peak metrics, i/dAUC and FFid measures do not always agree. This is expected in a setting where the reference peaks are only approximate comparators and where correlated biochemical features may allow the model to maintain classification performance after some regions are removed.

**Table 4:** *In Vitro* ’with DNA’ class performance vs DNA peaks: IoU (*↑*), PHR (*↑*); iAUC (*↑*), dAUC (*↓*), FFid+ (*↑*), FFid-(*↓*). Arrows indicate the direction of better performance.

Method	Level	IoU	PHR	iAUC	dAUC	FFid$^{+}$	FFid$^{-}$
GradientSHAP	Overall			0.48	**0.38**	**0.0470**	0.0322
	Class 0			**0.95**	0.83	**0.1181**	**−0.0295**
	Class 1	0.0000	0.0000	0.11	**0.05**	−0.0019	0.1147
	Class 2			0.28	**0.14**	**0.0133**	**0.0011**
Grad-CAM	Overall			0.52	0.59	0.0129	0.0031
	Class 0			0.74	0.71	0.0497	0.0066
	Class 1	**0.0094**	**0.3371**	0.17	0.36	−0.0117	0.0064
	Class 2			0.75	0.79	0.0015	0.0005
Spec-ReX	Overall			**0.63**	0.41	0.0037	**0.0007**
	Class 0			0.81	**0.52**	0.0101	0.0009
	Class 1	0.0008	0.0221	**0.37**	0.19	**0.0048**	**0.0024**
	Class 2			**0.78**	0.66	0.0003	0.0069

### Ex vivo results

The *ex vivo* experiments assess biomedical applicability rather than controlled validation. In these datasets there is no definitive attribution ground truth. Hence, the results only show how the methods behave when applied to clinically relevant tissue spectra, and whether the resulting attributions are consistent across model architectures and model classes.

***Oesophageal cancer results***

Supplementary Figs. F.6 and F.7 show the mean attribution maps obtained from the ResNet and custom CNN models. As in the preceding experiments, SHAP produces visually dense attribution maps, whereas Spec-ReX produces sparser responsibility maps. Grad-CAM gives broader attributions, reflecting the resolution limits of the convolutional layer used to generate the saliency map.

A notable finding is that the custom CNN and ResNet do not assign importance to the same spectral regions. This indicates that models trained for the same task can learn different decision strategies. Similar architecture-dependent feature use has been observed in computer vision^36^. In the present context, it is a reminder that attribution maps should be interpreted as explanations of a particular model, not as direct evidence of disease-associated spectral biomarkers.

Table 5 summarises the quantitative results. For the custom CNN, SHAP achieves the highest overall iAUC (0.74) and FFid$^{+}$ (0.0102), while Spec-ReX achieves the lowest overall dAUC (0.51) and FFid$^{-}$ (0.0023). At the class level, Spec-ReX achieves the lowest dAUC for both class 0 (0.72) and class 1 (0.02), while SHAP achieves the highest class 1 iAUC (0.41) and FFid$^{+}$ (0.0141). This pattern is consistent with SHAP identifying broader sets of sufficient features and Spec-ReX identifying sparser regions whose removal or preservation affects the classifier differently.

For the ResNet model, SHAP achieves the highest overall iAUC (0.74), lowest overall dAUC (0.56) and highest FFid$^{+}$ (0.0240), whereas Spec-ReX achieves the lowest overall FFid$^{-}$ (0.0045). Class-specific behaviour is again uneven. SHAP performs best for class 1 on iAUC, dAUC and FFid$^{+}$, while Spec-ReX has the lowest FFid$^{-}$. Thus, in the oesophageal dataset, the relative ranking of explainers depends on both the architecture and the metric.

Overall, the oesophageal results suggest two conclusions. First, the sensitivity–specificity pattern observed in the controlled experiments is still visible but less uniform in clinical tissue spectra. Second, architecture dependence is itself an important result: even when two models are trained on the same task, their learned spectral strategies may differ.

**Table 5:** Detailed results for Custom CNN and ResNet: Insertion AUC (iAUC *↑*), Deletion AUC (dAUC *↓*), and F-Fidelity (FFid$^{+}$*↑*, FFid$^{-}$*↓*). Best per metric in bold for each model.

Model	Method	Class	iAUC	dAUC	FFid$^{+}$	FFid$^{-}$
Custom CNN		Overall	**0.74**	0.52	**0.0102**	0.0085
	GradientSHAP	Class 0	0.94	0.79	**0.0077**	**−0.0050**
		Class 1	**0.41**	0.08	**0.0141**	0.0299
		Overall	0.59	0.64	0.0011	0.0065
	Grad-CAM	Class 0	0.82	0.76	0.0002	−0.0009
		Class 1	0.23	0.46	0.0024	0.0181
		Overall	0.69	**0.51**	0.0016	**0.0023**
	Spec-ReX	Class 0	**0.95**	**0.72**	0.0000	0.0002
		Class 1	0.29	**0.02**	0.0041	**0.0055**
ResNet		Overall	**0.74**	**0.56**	**0.0240**	0.0375
	GradientSHAP	Class 0	**0.95**	0.86	**0.0345**	**−0.0113**
		Class 1	**0.40**	**0.08**	**0.0078**	0.1150
		Overall	0.63	0.63	0.0020	0.0049
	Grad-CAM	Class 0	0.87	0.88	0.0023	0.0030
		Class 1	0.27	0.23	0.0014	0.0081
		Overall	0.69	0.58	0.0010	**0.0045**
	Spec-ReX	Class 0	0.94	**0.82**	0.0004	0.0039
		Class 1	0.26	0.19	0.0020	**0.0052**

***FTIR oral cancer results***

The oral FTIR dataset differs from the previous experiments in both modality and model type. Because the dataset is smaller, a PLS-DA model was used rather than DL. Grad-CAM is not applicable to this model, so Spec-ReX and SHAP are compared with VIP, a commonly used interpretability measure for latent-variable models.

Table 6 shows that SHAP and Spec-ReX perform similarly overall on insertion/deletion metrics. SHAP achieves the highest overall iAUC (0.70), while SHAP and Spec-ReX are tied for the lowest overall dAUC (0.43). VIP performs less well overall on both iAUC (0.62) and dAUC (0.49). Class-specific differences are more pronounced: SHAP performs best for class 0 by iAUC (0.79), whereas Spec-ReX performs best for class 1 by both iAUC (0.70) and dAUC (0.30).

The ROAR results are not discriminative. The overall *Δ*ACC$_{\text {ROAR}}$ confidence intervals include zero for VIP, SHAP, Spec-ReX and random masking. Random masking performs comparably to the XAI-guided masks. This indicates that, after retraining, the PLS-DA model can often recover performance from remaining spectral information. The ROAR results therefore cannot provide evidence of performance in this highly correlated spectral dataset.

**Table 6:** Insertion/Deletion and ROAR summary. Insertion/Deletion iAUC/dAUC as before; *Δ*ACC$_{\text {ROAR}}$ entries are the *overall and per-class means* across all $\alpha {>}0$ and across baselines (mean, zero, oppclass) with 95% CIs. We define *Δ*ACC *=* ACC(0) − ACC*(α )*, so positive values indicate an accuracy drop when masking (i.e., stronger evidence that masked regions matter).

Method	Class	iAUC (*↑*)	dAUC (*↓*)	*Δ*ACC$_{\text {ROAR}}$
VIP	Overall	0.62 [0.58, 0.67]	0.49 [0.47, 0.51]	−0.0157 [−0.0604, 0.0145]
	Class 0	0.75 [0.72, 0.78]	**0.46 [0.44, 0.48]**	−0.0347 [−0.1157, 0.0185]
	Class 1	0.49 [0.47, 0.52]	0.52 [0.50, 0.54]	0.0051 [−0.0101, 0.0202]
SHAP	Overall	**0.70 [0.66, 0.74]**	**0.43 [0.41, 0.45]**	−0.0145 [−0.0664, 0.0217]
	Class 0	**0.79 [0.76, 0.83]**	**0.46 [0.45, 0.48]**	−0.0324 [−0.1273, 0.0347]
	Class 1	0.61 [0.57, 0.65]	0.39 [0.36, 0.42]	0.0051 [−0.0101, 0.0202]
Spec-ReX	Overall	0.69 [0.67, 0.71]	**0.43 [0.39, 0.47]**	−0.0181 [−0.0652, 0.0133]
	Class 0	0.67 [0.64, 0.69]	0.56 [0.53, 0.58]	−0.0394 [−0.1227, 0.0185]
	Class 1	**0.70 [0.68, 0.73]**	**0.30 [0.28, 0.32]**	0.0051 [−0.0101, 0.0202]
Random	Overall	–	–	−0.0157 [−0.0652, 0.0194]
	Class 0	–	–	−0.0347 [−0.1250, 0.0324]
	Class 1	–	–	0.0051 [−0.0101, 0.0202]

Qualitative inspection of the heatmaps (Supplementary Fig. F.8) shows that VIP, SHAP and Spec-ReX agree most clearly around 1054–1062 $cm^{-1}$. This region has been associated with carbohydrate vibrations (C–O, C–C and ring vibrations) overlapping with phosphate contributions, which have been implicated in oral cancers^37^. This agreement is encouraging, but should be interpreted cautiously because no independent attribution ground truth is available.

The binary linear structure of PLS-DA also affects the attribution maps. VIP produces a single global importance profile by design. Spec-ReX and SHAP produce class-wise maps, but these maps are very similar across classes. For SHAP, one class attribution map is the inverse of the other. This reflects the fact that, in a binary linear classifier, the same features define the decision boundary for both classes, while the class label depends on which side of the boundary a spectrum falls. SHAP therefore provides directional information in addition to feature importance, but the preceding *in silico* experiments show that such additional information may also include spurious signal.

Overall, the oral FTIR results show that both SHAP and Spec-ReX can be applied to a simple linear spectral classifier and can produce plausible attribution maps. Although VIP is an interpretability framework as opposed to an XAI method, it performs relatively poorly compared to both SHAP and Spec-ReX. This is consistent with literature showing that VIP can be inconsistent and the threshold choice is arbitrary^38^.

## Discussion

This study evaluates Spec-ReX across a progression from controlled synthetic spectra to real tissue spectra. The main result is not that one XAI method is universally superior. Rather, the experiments show that different XAI methods have different failure modes, and that these differences become more consequential as ground truth becomes less certain.

The definitive *in silico* experiment provides the clearest evidence of explainer fidelity. Because the discriminative peaks were known by construction, IoU and concentration can be interpreted directly. Under these conditions, Spec-ReX recovered the relevant regions while producing little attribution mass outside them. SHAP also identified relevant peaks but gave more diffuse attributions, and Grad-CAM was limited by the spatial resolution of the final convolutional layer. This directly supports the interpretation that Spec-ReX is comparatively specific, whereas SHAP is comparatively sensitive.

The ambiguous *in silico* and *in vitro* experiments show why assessing spectral XAI is more difficult in realistic settings. DNA-associated peaks provide a biochemical reference, but not a definitive ground truth. A model may learn only a subset of the DNA signal, and real spectra can include peak shifts, conformational effects and correlated features. In this setting, disagreement between IoU, PHR, insertion/deletion AUC and F-Fidelity reflects a limitation of current XAI metrics for highly correlated spectral data. Measures that reward sensitivity can favour diffuse methods, while measures that reward specificity can favour sparse methods.

The *ex vivo* datasets should be interpreted most cautiously. They are clinically relevant but lack a known ground truth. The oesophageal results show that different model architectures trained on the same task can rely on different spectral regions. This does not imply that the XAI methods are unfaithful, but that the learned decision strategies themselves may be model-dependent. Faithful explanations of shortcut-driven models are useful because they can reveal model brittleness. Such explanations should not be treated as evidence of disease biology, but of what features a model has learnt to drive a classification.

Clinical usefulness is a separate question from XAI fidelity. Sparse explanations may be easier for spectroscopists or clinicians to inspect, but sparsity alone does not guarantee usefulness. Future studies should include domain experts and assess whether attribution maps improve calibration of trust, identification of model failure, and actionability in realistic diagnostic workflows. Such studies could draw on existing frameworks for evaluating domain relevance, coherence and utility of clinical XAI outputs^10,20^.

Spec-ReX remains under active development. Methods to improve the fidelity of the tool include more refined mask generation. This may include the use of generative models to construct realistic class-neutral spectra, although this would likely increase computational time. A more tractable method applicable to time-dependent waveforms is to occlude in the Fourier domain, allowing for more precise feature removal^39^. Whether this is applicable to vibrational spectra is an open question. It may also be possible to make existing architectures more amenable to Spec-ReX. Activation-deactivation is a method which applies the masking of features by acting directly on the model weights^40^. These approaches may reduce the need to choose a neutral masking value and improve the realism of perturbations.

Spec-ReX is implemented here as a feature-attribution method, similar to SHAP and Grad-CAM. However, the full theory of ReX also allows the construction of counterfactual explanations. Counterfactuals have the advantage of a precise formal definition and can identify an approximately minimal subset of features sufficient for a classification^41^. This may be valuable for model developers, particularly when comparing architectures, but it remains unclear how best to aggregate instance-level counterfactual explanations into class-wise explanations that are useful to clinicians.

## Conclusion

This paper assessed the fidelity of Spec-ReX against common XAI techniques across synthetic, biochemical and clinical vibrational spectroscopy datasets. In the definitive *in silico* setting, where attribution ground truth was known, Spec-ReX produced the most concentrated and specific explanations. In ambiguous and clinical settings, the results were more nuanced: SHAP often behaved as the more sensitive method, while Spec-ReX tended to return sparser responsibility maps.

These findings support Spec-ReX as a competitive method for explaining spectral classifiers, but they do not imply that any single XAI method is best for all applications. Nor do they imply that highlighted spectral regions are biologically causal. The explanations are faithful to the trained model under the specified perturbation scheme. This distinction is central for clinical spectroscopy, where explanations may be most useful not as proof of disease mechanisms, but as tools for identifying which features drive classification.

The study also highlights the difficulty of evaluating XAI when the ground truth is unavailable. Future work should develop fidelity metrics tailored to correlated spectral data and should test whether sparse responsibility maps improve expert interpretation in user-centred studies. Clinical utility remains to be validated.

## Supplementary Information

Supplementary Information.

## Data Availability

In Silico code to reproduce data available at: GitHub repository (https://github.com/NBlake/Spec-ReX-XAI-for-vibrational-spectroscopy/tree/main) In Vitro dataset available at: Zenodo record 17938435 (https://zenodo.org/records/17938435) The Oesophageal Cancer dataset is available at: Zenodo record 10405264. (https://zenodo.org/records/10405264) The FTIR Oral Cancer Dataset is available from the corresponding author upon reasonable request.
